# “Inverted Snowing-Cloud” Sign in Endogenous Candida Endophthalmitis

**DOI:** 10.18502/jovr.v17i2.10807

**Published:** 2022-04-29

**Authors:** Pasha Anvari, Reza Mirshahi, Ahad Sedaghat, Khalil Ghasemi Falavarjani

**Affiliations:** ^1^Eye Research Center, The Five Senses Institute, Rassoul Akram Hospital, Iran, University of Medical Sciences, Tehran, Iran

**Keywords:** Candida, Endogenous Endophthalmitis, Optical Coherence Tomography

## Abstract

*Candida* spp. is the most common cause of endogenous fungal endophthalmitis. The diagnosis of this rare disease is based on clinical findings supported by positive blood culture. Recently, it has been shown that optical coherence tomography (OCT) characteristic findings are beneficial in making a correct diagnosis of fungal infection in cases with endogenous endophthalmitis. The current photo-essay aims to highlight the role of OCT in diagnosis of *Candida* endogenous endophthalmitis where OCT imaging of one of the retinal lesions disclosed a pre-retinal hyper reflective lesion with overlying punctate vitreous opacities. We propose “inverted snowing-cloud” sign for this OCT pattern considering the resemblance of the vitreous opacities to snowflakes.

**Figure 1:**
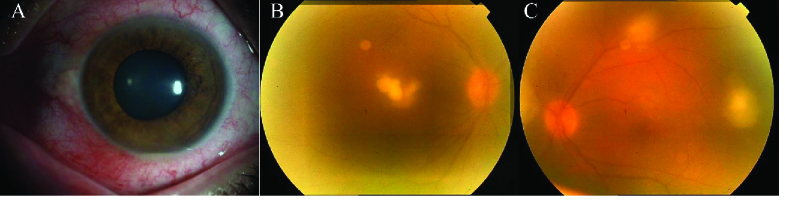
Slit photo of the left eye demonstrating minimal conjunctival injection despite the formation of the hypopyon in the anterior chamber (A). Fundus photo of both eyes showing multiple fluffy pre-retinal yellowish lesions with the involvement of the macular in the right eye (B & C).

**Figure 2:**
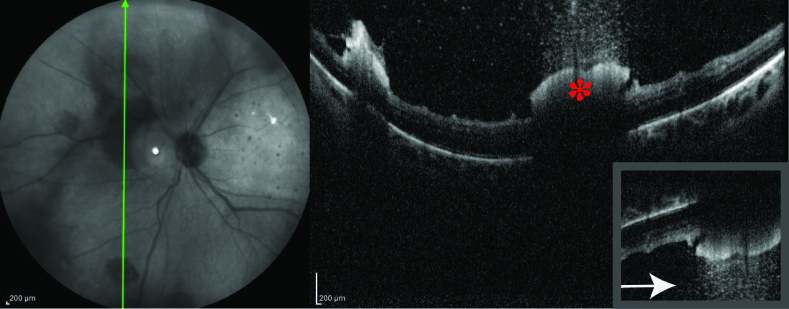
Optical coherence tomography (OCT) of one of the lesions in the left eye shows hyperreflectivity of the inner retina (asterisk) with posterior shadowing (“rain-cloud” sign) alongside vitreous aggregates extending to the vitreous cavity. In the inset, by inverting the image, the resemblance to a “snowing-cloud” becomes more evident with vitreous aggregates representing snowflakes (Asterisk).

##  Financial Support and Sponsorship

Nil.

##  Conflicts of Interest

There are no conflicts of interest.
